# Accidental Gluten Contamination in Traditional Lunch Meals from Food Services in Brasilia, Brazil

**DOI:** 10.3390/nu11081924

**Published:** 2019-08-16

**Authors:** Priscila Farage, Renata Puppin Zandonadi, Lenora Gandolfi, Riccardo Pratesi, Ana Luísa Falcomer, Letícia Santos Araújo, Eduardo Yoshio Nakano, Verônica Cortez Ginani

**Affiliations:** 1 Faculty of Nutrition, Federal University of Goiás (UFG), Campus Colemar Natal e Silva, Rua 227 qd.68 s/n, Setor Leste Universitário, Goiânia 74605-080, Brazil; 2 Department of Nutrition, Faculty of Health Sciences, University of Brasilia (UnB), Campus Darcy Ribeiro, Asa Norte, Brasilia 70910-900, Brazil; 3 Faculty of Medicine, University of Brasilia (UnB), Campus Darcy Ribeiro, Asa Norte, Brasilia 70910-900, Brazil; 4 Department of Statistics, Central Institute of Sciences, University of Brasilia (UnB), Campus Darcy Ribeiro, Asa Norte, Brasilia 70910-900, Brazil

**Keywords:** celiac disease, gluten, gluten-free, contamination, food contamination, foodservice

## Abstract

This study aimed to evaluate the occurrence of gluten contamination in naturally gluten-free meals from food services in the Federal District, Brazil. This is an exploratory cross-sectional quantitative study in which a total of 180 samples of naturally gluten-free dishes were collected from 60 food services in Brazil. The enzyme-linked immunosorbent assay was used for the quantification of gluten. As established by the Codex Alimentarius, the threshold of 20 ppm of gluten was considered as the accepted upper gluten level for gluten-free food. A total of 2.8% (95% CI: 0.3–5.2%) gluten contamination was found in the samples. Among the 60 food services, 6.7% (95% CI: 2.7–10.6%) displayed at least one contaminated food in our sample. The occurrence of gluten contamination in naturally gluten-free preparations was uncommon and low on a quantitative basis.

## 1. Introduction

Celiac disease (CD) is an immune-mediated enteropathy disorder triggered by the consumption of gluten in genetically predisposed individuals, which affects about 1% of the worldwide population [1]. The immune reaction triggered by gluten leads to small bowel inflammation and villous atrophy [2]. Standard intestinal features of CD include chronic diarrhea, weight loss, abdominal pain and bloating, constipation, and flatulence; non-classical extraintestinal features comprise anemia, osteoporosis, neurological disturbances, fatigue, and depression [1,3,4,5]. Despite many studies in the field, the only safe and effective treatment for CD so far is the complete exclusion of gluten from the diet [6].

Besides CD, there are other health conditions related to gluten that require a gluten-free diet (GFD). The gluten-related disorders (GRD) comprise three primary forms of gluten reactions: allergic (wheat allergy), autoimmune (CD, dermatitis herpetiformis, and gluten ataxia), and possibly immune-mediated (gluten sensitivity) [1]. These conditions combined affect about 10% of the population [7]. GRDs have not yet been fully elucidated, and further studies are needed to clarify whether the spectrum of toxic cereals, gluten intake threshold, and disease duration are similar to CD. Currently, as well as in CD, the treatment for the GRDs is the lifelong exclusion of gluten from the diet [1].

In CD individuals, adherence to the GFD results in a remission of symptoms, normalization of serum autoantibodies typical of the disease, and recovery of intestinal histological architecture [8]. However, signs of inflammation persist in approximately 50% of patients, and this is probably related to inadvertent exposure to gluten [9].

The presence of gluten traces in food may be enough to cause health problems in CD. In a systematic review, authors found that some patients tolerate an average of 34–36 mg of gluten per day. However, others develop mucosal abnormalities while consuming 10 mg of gluten per day. Therefore, the authors mention that although there is no evidence to recommend a single definitive threshold, a daily gluten intake of less than 10 mg is unlikely to provoke significant histological alterations [10].

As proposed by the Codex Alimentarius, in the gluten-free foods (GFF), the gluten level cannot exceed 20 ppm (mg/kg) in total [11]. Considering this allowed limit, a total of 500 g of GFF containing 20 ppm of gluten would be permitted in the GFD without extrapolating the safe daily gluten intake of 10 mg. Nevertheless, most countries lack a consistent monitoring process to evaluate gluten content in supposedly GFF [12].

Hence, eating products from the food industry or food from restaurants may represent a risk for CD patients [13,14]. The presence of gluten in supposedly GFF may be a result of cross-contamination caused by shared production areas, kitchenware not sanitized properly, and inadequate procedures by the restaurant staff [14].

Some studies have investigated the occurrence of gluten contamination in processed food. In Italy, the authors found a 9% rate of contamination in gluten-free products available in the Italian market [8]. In Brazil, studies have identified gluten contamination varying from 11% to 12.9% in industrialized products [15,16,17]. In the United States, gluten contamination above 20 ppm was detected in 20.5% of foods labeled “gluten-free” in the study by Lee, Anderson, and Ryu (2014) [18]. In the pilot study by Thompson et al. (2010), a 32% contamination rate was detected among a sample of 22 inherently gluten-free grains, seeds, and flours not labeled gluten-free (single-ingredient foods such as millet) [19]. In contrast, Sharma, Pereira, and Willians found that gluten-free labeled foods had 98.9% gluten-free labeling compliance in a market survey conducted in the US (only three samples out of 275 had >20 ppm of gluten) [20]. In food services, there are even fewer data regarding gluten contamination in food. Oliveira et al. [21] identified the presence of gluten in 16.7% of bean samples from self-service restaurants in Brazil, which is a naturally GFF [21]. A study conducted in Ireland found 10% contamination in GFF obtained from catering outlets [22].

Besides preventing histological alterations and clinical manifestations of CD, the GFD is crucial for reducing the risk for long-term complications [23]. However, it is important to take into account that the need to adopt a GFD compromises the quality of life in patients with CD. Data from the study of Biagetti, Naspi, and Catassi [24] reveal that poor awareness of CD treatment in catering staff and the low availability of GFF in most restaurants and cafeterias, among other factors, could be related to the difficulties patients experience in coping with the GFD [24].

The recognition of CD as a public health problem has increased nowadays [25] and, in this context, the assessment of GFF safety in places commonly frequented by the population which are available for CD patients eating outside home must be emphasized. Therefore, this study aimed to evaluate the occurrence of gluten contamination in naturally gluten-free meals from food services in the Federal District, Brazil.

## 2. Materials and Methods

### 2.1. Sampling

According to the Brazilian resolution n° 216 from the National Health Surveillance Agency, “food services” include establishments such as canteens, buffets, confectioners, industrial kitchens, institutional kitchens, delicatessens, snack bars, bakeries, pastry shops, restaurants, rotisseries, and similar items [26]. For the selection of establishments in this study, three main databases of food services from the city of Brasilia were used. The Regional Nutrition Council provided a record of restaurants. A list of the city’s daycare centers, schools, nursing homes, and philanthropic institutions assisted by the food and nutrition security program *Mesa Brasil*, which is a national network of food banks that aim to combat hunger and food waste, was also used. At last, a list of the government community restaurants was provided by the Subsecretariat for Food and Nutrition Security of the state.

A total of 282 establishments were obtained considering these three databases. These food services comprise restaurants where Brazilians commonly have lunch, including commercial restaurants, governmental restaurants, and institutional restaurants (located in schools, daycares, and nursing homes). Those are regular establishments that produce both gluten-containing food, such as pasta and pies, for example, and naturally GFF, such as rice, meat, beans, and salads, among other items.

Food services were invited to participate in the study by telephone and/or email contact, and a total of 60 establishments agreed to take part. This sample size allowed estimating a 95% confidence interval of the prevalence of gluten contamination with an error of estimation of 4%.

It is important to clarify that control of gluten cross-contamination in restaurants is not a regular practice in Brazil. The Brazilian legislation does not require cross-contamination prevention in food services. Furthermore, it does not set a limit of gluten content for food considered gluten-free. In Brazil, all food that is naturally gluten-free or produced with gluten-free ingredients can be identified as “gluten-free” without the need for laboratory analysis. Therefore, refusal to participate in the study was not believed to be related to fear of identification of contaminated samples in the establishment, since this is not a concern of nutritionists and owners of food services in general. Common reasons to deny participation in the study were overloaded routine and lack of available time to accommodate the researchers.

The Ethics Committee of the Faculty of Health Sciences of University of Brasilia approved the study protocol (protocol number: 60987816000000030). The person in charge of each food service signed an agreement term for authorization of the study in the establishment.

### 2.2. Collection of Food Samples

Food services were visited during the usual production period of lunch between January and November 2017. The day menu was requested to the chef or nutritionist responsible for the establishment. Since the labeling of gluten-free foods in restaurants is not required by legislation in Brazil, the majority of food services did not classify menu dishes in regards to gluten content. Researchers evaluated the menu in each establishment on the day of the visit and identified traditional and inherently gluten-free dishes (such as rice, beans, meats, and vegetables). Moreover, the label of all the packaged ingredients used to prepare those dishes was checked (such as oil, sauces, and seasoning). Foods selected for collection were chosen randomly among those naturally gluten-free dishes. A total of three different samples (different food items) were collected in each food service.

Since the study aimed to detect accidental gluten contamination in naturally GFF, all the ingredients used in the food samples selected were checked by verification of the label in order to ensure that no intentional gluten-containing ingredient was used in the food collected. In case any gluten-containing ingredient was used in a naturally gluten-free dish—for example, wheat flour to thicken a meat sauce—this dish was not considered for the collection process, since it would obviously present gluten in its composition.

In all the establishments, gluten-free and gluten-containing food were produced in the same production area, as checked by the researcher. Three food samples were collected in each establishment, generating a total of 180 samples, categorized according to the type of food in: (i) main course (meats and vegetarian protein options—52 samples); (ii) garnish (menu item accompanying the main course, including braised vegetables, soufflé, pies, “farofa”, French fries, and others—55 samples); (iii) side dishes (dishes culturally daily consumed by the Brazilian population, such as rice, beans, and their variations—71 samples); and (iv) others (two samples).

Foods were categorized into three types because the lunch meal in Brazil is traditionally composed mainly by these dishes (main course, garnish, and side dishes). In cases where there was no gluten-free food alternative from the above categories, another gluten-free dish of the day was also randomly selected (category “others”) such as salads (one sample) and desserts (one sample). Bakery/bread/pizza and dairy are not usually consumed at lunch in Brazil. Although desserts may be present in the lunch meal, not all establishments serve dessert on a daily basis.

### 2.3. Handling of Food Samples

Handling of samples was conducted at the Laboratório de Técnica Dietética, University of Brasilia, Brazil. Before processing the samples, laboratory surfaces, instruments, and equipment were cleaned with water, regular detergent, Triton X 5% solution (a detergent used to remove proteins such as gluten), distilled water, and ethanol solution (70%) in order to prevent any gluten contamination from the environment. This procedure was also performed between the homogenization processes of each sample, to prevent contamination among them, as proposed by Silva [15]. Samples were homogenized (each entire food sample was homogenized—100 g) using a food processor with detachable external parts that were cleaned after every run. Samples were coded by numbers and kept frozen until the date of the extraction step. Samples’ details (origin, type of food, and ingredients) were compiled in an excel spreadsheet. In the following steps of the analysis (extraction and ELISA), samples were identified only by number. Therefore, analysts were not aware of what samples were being analyzed.

### 2.4. Laboratory Analysis

Gluten content of samples was determined by the Ridascreen Gliadin sandwich R5 enzyme-linked immunosorbent assay R-7001 (R-Biopharm, Darmstadt, Germany) at the Laboratório Interdisciplinar de Biociências (Laboratório de Pesquisas em Doença Celíaca), University of Brasília, Brazil. The manufacturer’s instructions were strictly followed, as described below.

For the extraction of gluten from the food matrix, the extraction cocktail solution (R7016) from R-Biopharm was used (Darmstadt, Germany). One test portion of 0.25 g of each homogenized sample was weighed and mixed with 2.5 mL of the cocktail in pre-labeled falcon tubes. Samples were incubated for 40 min at 50 °C (122 °F). After being cooled down, samples were mixed with 7.5 mL of 80% ethanol and then shaken by a rotator at room temperature for 1 h. Then, samples were centrifuged at 2500 g for 10 min, and the supernatants were transferred to other falcon tubes and stored at room temperature in the dark until the date of the ELISA step, which was performed in one to two days after the extraction step, respecting the 8-week sample viability period.

For the ELISA step, extracted samples were diluted with diluted sample diluent at 1:12.5. All of the next steps were automatically performed by the Best 2000^®^ (BioKit S.A., Werfen, Barcelona, Spain) automated microplate immunoanalyzer. An amount of 100 μL of each standard solution, samples, and controls were added to separate triplicate wells and incubated for 30 min at room temperature. Three washing steps were performed using the washing buffer. An amount of 100 μL of the diluted enzyme conjugate was added to each well, followed by an incubation of 30 min at room temperature, and again three washing steps. Then, 50 μL of the substrate and 50 μL of chromogen were added to each well, followed by an incubation period of 30 min at room temperature in the dark. At last, 100 μL of the stop reagent was added to each well, and the absorbance was measured at 450 nm within 30 min after the addition of the stop solution.

Results were calculated using a cubic spline function, as suggested by the manufacturer of the ELISA kit. The lower limit of quantification of the kit is 2.5 ppm (mg/kg) of gliadin, corresponding to 5 ppm (mg/kg) of gluten. As established by the Codex Alimentarius, the threshold of 20 ppm of gluten was considered as the accepted upper gluten level for GFF [11]. Foods containing more than 20 ppm of gluten were considered contaminated.

For quality control of the extraction step, the test implementation, and the handling of the Ridascreen Gliadin (R7001), the processed Gliadin Assay Controls (R7012) from R-Biopharm were used.

### 2.5. Statistical Analysis

For data analysis, the GraphPad Prism, version 6 (San Diego, CA, USA), and the Microsoft Excel, version 2013 (Redmond, WA, USA), software were used. The number of meals served daily at lunch were presented as mean ± standard deviation (SD). The confidence intervals of the prevalence of gluten contamination in food samples were obtained by the normal approximation. In the food services, the confidence intervals also considered the correction for finite populations.

## 3. Results

A total of 60 food services agreed to participate in this study. The characteristics of the food services are summarized in Table 1.

Institutional restaurants include the food services of government agencies, refectories of philanthropic institutions, and refectories for employees at industries, hospitals, and hotels in the city. These establishments accounted for 30% of the sample sites. It is noteworthy that the number of meals served daily at lunch is high (1076 ± 1807) at these food services, since a large portion of the population does not return home for this meal and consequently eats at the workplace.

Daycare centers and school canteens primarily serve children and adolescents, but also serve the local staff. These establishments accounted for 30% of the sample. The number of meals served daily at lunch is 234 ± 148.

Community restaurants represented 23.3% of the sample sites. These establishments are part of a government assistance program aimed at guaranteeing access to food for the low-income population [27,28]. Community restaurants provide nutritional quality meals at a low-cost [27]. The number of meals served daily at lunch is high (1491 ± 324), as the initiative of the community restaurants seeks to reach a large portion of the population that would not be able to obtain food otherwise.

Commercial restaurants accounted for 16.7% of the study sample. These establishments are distributed throughout the city, and customers who attend these locations have different profiles, varying from children to the elderly. These food services have a self-service system in which the customers pick as much as they want from a wide range of dishes. The customer’s plate is weighed and the price paid for the meal is proportional to the amount of food. This type of establishment is common in Brazil. The number of meals served daily at lunch is 233 ± 105.

**Figure 1:**
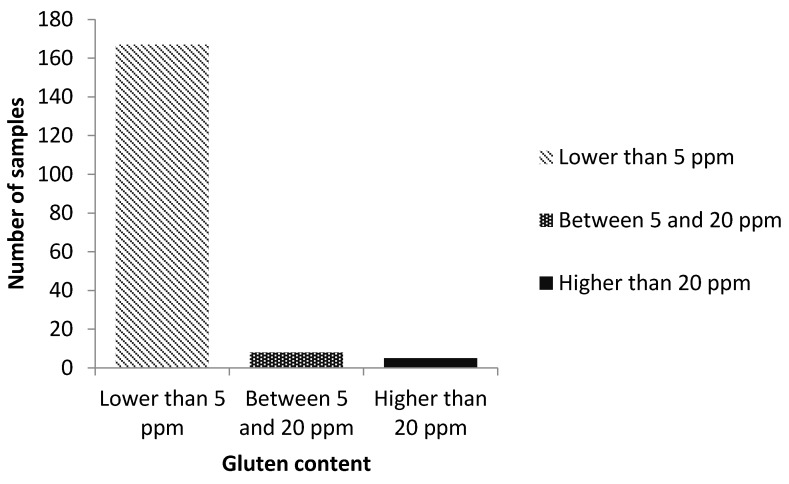
Distribution of gluten concentration of the collected samples.

The distribution of gluten concentration of the collected samples are shown in Figure 1. Among the 180 samples, a total of five samples were contaminated with gluten (2.8%; 95% CI: 0.3–5.2%). Gluten content was under the quantification limit (5 ppm) in 92.8% (95% CI: 89.0–96.6%) of the samples analyzed. A total of eight samples (4.4%; 95% CI: 1.4–7.5%) displayed gluten content between 5–20 ppm.

Among the contaminated samples, two were from the same establishment. Therefore, out of 60 food services, four displayed contaminated foods (6.7%; 95% CI: 2.7–10.6%). The contaminated samples were: braised pumpkin and okra (garnish); roast beef, grilled steak, and Provençal chicken (main course); and black bean (side dish). These samples came from the following types of food services: an industry refectory; a school canteen; a commercial restaurant; and a community restaurant (two contaminated samples). When analyzing contamination by type of food, the main course was the category with the highest percentage of contamination among its samples, though it was still relatively low (5.8%), followed by garnish (1.8% contamination) and side dishes (1.4% contamination). Among the five contaminated samples, four displayed gluten concentration above the limit of quantification of the ELISA test (80 ppm), and the other sample presented 22.8 ppm of gluten.

## 4. Discussion

Most of the foods Brazilians eat at lunch are naturally GFF, such as rice, beans, salad, meats, and vegetables [29]. Therefore, it is expected that regular food services would provide viable choices for CD patients. However, foods such as pasta, soufflé, pies, breaded food, and other gluten-containing dishes are also usually prepared at food services, which could lead to cross-contamination of GFF. Studies point out that most celiac individuals feel restricted eating out in restaurants for fear of being inadvertently exposed to gluten [30,31,32].

Although restaurants from our sample have nutritionists who are technically responsible for the establishment and quality of the meals served, this did not guarantee the absence of gluten in all the foods, which indicates the need for supervision and better guidance of employees in regards to food preparation procedures.

It is important to emphasize that gluten-contamination control is not a regular practice in food services from Brazil. The Brazilian legislation does not require cross-contamination prevention in meals from restaurants [15]. A study conducted by Laporte and Zandonadi [33] showed that chefs from restaurants in Brazil are not aware of foods that contain gluten, nor of adequate procedures when preparing gluten-free food in order to avoid cross-contamination [33]. Therefore, the study visits were not seen by staff as a monitoring assessment, and employees probably did not alter their regular practices due to the research on the visit day.

Our sample was composed of different types of food services that attend to diverse publics. Thus, these establishments cover various possibilities of lunch options for celiac individuals, contemplating profiles of different age groups and socio-economic conditions, among other aspects.

Gluten contamination above 20 ppm was present in 2.8% of the foods sampled. The occurrence of gluten contamination is lower than the one found by Oliveira et al. [21] in 60 bean samples from self-service restaurants in Brazil (16% of contamination). However, in that study, the ingredients used for the preparation of beans were not investigated by the researchers. Therefore, the intentional use of wheat flour may have occurred in order to thicken the broth—a common practice in Brazilian restaurants, which jeopardizes the treatment of celiac individuals who may not be aware of the presence of gluten in this type of food [21]. A total of 28 bean samples were evaluated in the present study. Of those, one displayed gluten content above 20 ppm. Since beans are a typical dish of the Brazilian culture, contamination in this type of food may affect a large part of CD patients.

In another study from Brazil, results revealed an occurrence of 21.5% of contamination in bakery products. Nevertheless, a higher percentage of contamination is expected in this type of establishment, since there is an extensive manipulation of wheat flour for the production of bread, cakes, biscuits, and similar, which increases chances of cross-contamination [34].

In regard to the type of food analyzed, out of the five contaminated food samples, three were main courses. One of these samples—Provençal chicken—is served with sauce. In the study by Simpson and Thompson [35], the authors warned that sauces and dressings might be a source of gluten contamination in restaurants. The other main courses were roast beef and grilled steak, which would be theoretically safer for CD individuals, since those are meat-based dishes that contain fewer ingredients. However, a cleaned section of the grill is also recommended for the prevention of gluten cross-contamination in restaurants [35]. Braised pumpkin and okra (garnish) and black bean (side dish) also displayed gluten content above 20 ppm, each representing 20% of the contaminated samples. Since the number of contaminated samples in this study was low, it is not possible to evaluate whether the risk of contamination is significantly higher or lower in any food category.

The correct information about the presence/absence of gluten in food provided by food services is very important for those individuals who need to maintain a GFD. In a systematic review, 24 cross-sectional studies that assessed gluten-contamination in gluten-free industrial and non-industrial products were evaluated. Results from the statistical meta-analysis pointed out a contamination prevalence of 13.2% (95% CI: 10.8–15.7%) in industrial products and 41.5% (95% CI: 16.6–66.4%) in non-industrial food products. Industrial products labeled as “gluten-free” showed a lower percentage of gluten contamination in comparison to non-industrialized products. Despite that, any contaminated sample found in labeled “gluten-free” industrial food presents greater relevance, since it indicates that the label may be unreliable, and these “gluten-free” products should not be considered safe for celiac patients [36]. In Brazil, there is an urgent need to strengthen legislation on gluten-free products, as there is no obligation to identify restaurant meals in regard to the presence of gluten, which compromises the adoption of an adequate GFD even more.

As advocated by the Codex Alimentarius, GFF must be prepared with special care under Good Manufacturing Practices (GMP) in order to avoid gluten cross-contamination [11]. In the study by Bicudo, standardized operating procedures (SOPs) were developed and implemented for the prevention of cross-contamination in a bakery that produced both gluten-containing and gluten-free products. The authors applied a GMP checklist and corrective actions for the problems encountered. The elaboration of SOPs along with measures regarding the GMP was effective in the control of gluten contamination at the site studied [37].

In Brazil, a checklist specifically elaborated for the prevention of gluten cross-contamination in food services has been created, and it is currently undergoing a process of validation [38].

Data collected in the study by Petruzzelli et al. [39] also revealed the appropriateness of the Hazard Analysis and Critical Control Point (HACCP) system to reduce the risk of gluten contamination in a school catering facility. The authors found only one sample out of 87 with gluten content above 20 ppm (1.2%). However, in this case, the facility displayed a separated specific area for the preparation of special diets, which was endowed with specific cooking tools, pans, equipment, and storage closets and, for the preparation of lactose-free and gluten-free meals, dedicated burner stoves were used [39].

In the present study, gluten-containing and gluten-free meals were produced in the same facility area, without separation of routines, utensils, and equipment. Even so, the occurrence of gluten contamination was not as high as expected. As a matter of fact, it was quite low. The majority of the samples displayed gluten content below the quantification limit. It is important to mention that there is a big concern regarding gluten cross-contamination in the nutritional assessment of CD patients. These individuals are regularly advised on this subject, and it is a topic often discussed in the scientific literature [12,35,40].

Nevertheless, it is noteworthy that staff in food services is usually not well educated in regards to the subject of CD and gluten [30,31,33]. A Canadian survey conducted with 5912 celiac participants revealed that one of the difficulties experienced by patients is the concern about cooks in restaurants not being trained in preparing gluten-free meals. Participants talked about not being able to eat at restaurants, because the food may be contaminated with gluten [41].

Although the treatment for CD could be considered relatively simple from a medical point of view since it does not require regular use of medication and/or medical intervention procedures, it brings a lot of social compromise to the patient’s life [31,42,43,44]. Quality of life is negatively affected for many reasons, especially since treatment entails a demanding, lifelong, restrictive diet [45] subject to the risk of contamination.

The need to avoid gluten-containing food often brings considerable changes in eating patterns and lifestyle that may be difficult to maintain over a lifetime [41]. Thus, it is important to reflect on how much the patient should be burdened with issues related to contamination. That does not mean that contamination should be underestimated. It is well known that the presence of gluten traces in GFF may prevent the histological and clinical recovery of patients with CD [46]. However, more studies are necessary to evaluate this matter more carefully.

Future research should focus on identifying categories of foods and types of food services that are more susceptible to the occurrence of cross-contamination and what inadequate procedures and practices indeed lead to a contaminated final product or meal.

Furthermore, long working hours typical of the routine of many individuals nowadays make it hard for people to return home for lunch, which leads to an increasing trend of having meals in establishments such as self-service and fast-food restaurants [21]. Therefore, it is important to raise awareness about CD and gluten among food services. Staff should be regularly trained in order to minimize the risk of producing contaminated food.

The highlights of this study include the sample being composed of a great diversity of types of food services that may be frequented by CD patients, which enriches the data obtained. Moreover, there are very few data in the literature regarding gluten cross-contamination in meals from food services. However, it is important to mention the limitations of the research, such as the size of the sample of restaurants (n = 60) due to the low response rate among contacted food services to participate in the study, and also the difficulties regarding the comparison of results due to the lack of similar studies. In addition to the number of restaurants, depending on the homogenization and distribution of gluten after sample preparation, the sample size (0.25 g) used in ELISA may not be enough to measure gluten in the meal, especially when only one test portion was analyzed. This limits the assessment of variability between gluten measurements within a sample. Moreover, only three food samples from each site were collected. It is possible that other foods that were not analyzed in this study had gluten content above the allowed limit.

Another limitation refers to the analytical method used. Although it is accepted as the gold-standard for gluten analysis by the Codex Alimentarius and the Association of Official Analytical Chemists (AOAC), the ELISA R5 technique requires at least two epitopes in the protein. Nevertheless, proteins are fragmented during food processing and may be converted into peptides in which only one toxic epitope appears, which might compromise part of the results [47].

## 5. Conclusions

Although there is much concern regarding the presence of gluten traces in supposedly GFF, the occurrence of contamination found in meals from food services in this study was low. In a certain way, this creates the prospect of reliability for celiac patients when eating out-of-home in Brazil. However, this issue must be carefully assessed.

Adherence to the GFD is essential for the clinical, histological, and serological recovery of patients with CD and consequent improvement in the quality of life. Despite the primarily dietary treatment, people with CD face several difficulties. In this context, the fear of being involuntarily exposed to gluten through the consumption of contaminated food stands out.

Despite the findings from this study, an effort should be made by food services and also the food industry in order to minimize the risk of cross-contamination in food. That would create a more reliable scenario for CD patients who need to eat out-of-home. Furthermore, future research should focus on identifying inappropriate procedures that cause gluten-contamination and proposing new strategies to overcome this issue.

## Figures and Tables

**Table 1:** Characterization of the Food Services of the study (*n* = 60).

Type	Percentage (%)	Number of Meals Served Daily at Lunch per Restaurant by Type of Food Service (Mean/SD *)
Institutional restaurants	30	1076 ± 1807
Day care centers/School canteens	30	234 ± 148
Community restaurants	23	1491 ± 324
Commercial restaurants	17	233 ± 105

* Standard Deviation.

## References

[1] Sapone A., Bai J.C., Ciacci C., Dolinsek J., Green P.H.R., Hadjivassiliou M., Kaukinen K., Rostami K., Sanders D.S., Schumann M. (2012). Spectrum of gluten-related disorders: Consensus on new nomenclature and classification. BMC Med..

[2] Fasano A., Araya M., Bhatnagar S., Cameron D., Catassi C., Dirks M., Mearin M., Ortigosa L., Phillips A. (2008). Federation of International Societies of Pediatric Gastroenterology, Hepatology, and Nutrition Consensus Report on Celiac Disease. J. Pediatr. Gastroenterol. Nutr..

[3] Barker J.M., Liu E. (2008). Celiac disease: Pathophysiology, clinical manifestations, and associated autoimmune conditions. Adv. Pediatr..

[4] Woodward J. (2007). Coeliac disease. Medicine.

[5] Rivera H. (2013). Gluten: Food Sources, Properties and Health Implications.

[6] Kaukinen K., Lindfors K. (2015). Novel treatments for celiac disease: Glutenases and beyond. Dig. Dis..

[7] Sapone A., Lammers K.M., Mazzarella G., Mikhailenko I., Cartenì M., Casolaro V., Fasano A. (2010). Differential Mucosal IL-17 Expression in Two Gliadin-Induced Disorders: Gluten Sensitivity and the Autoimmune Enteropathy Celiac Disease. Int. Arch. Allergy Immunol..

[8] Verma A.K., Gatti S., Galeazzi T., Monachesi C., Padella L., Del Baldo G., Annibali R., Lionetti E., Catassi C. (2017). Gluten contamination in naturally or labeled gluten-free products marketed in Italy. Nutrients.

[9] Shamir R., Heyman M.B., Koning F., Wijimenga C., Gutierrez-Achury J., Catassi C., Gatti S., Fasano A., Discepolo V., Korponay-Szabó I.R. (2014). Celiac Disease. J. Pediatr. Gastroenterol. Nutr..

[10] Akobeng A.K., Thomas A.G. (2008). Systematic review: Tolerable amount of gluten for people with coeliac disease. Aliment. Pharmacol. Ther..

[11] Codex Alimentarius Comission (2008). Draft Revised Standard for Foods for Special Dietary Use for Persons Intolerant to Gluten. http://www.jhnfa.org/CCNFSDU07.pdf.

[12] Diaz-Amigo C., Popping B. (2012). Gluten and gluten-free: Issues and considerations of labeling regulations, detection methods, and assay validation. J. AOAC Int..

[13] Farage P., Zandonadi R.P. (2014). The Gluten-Free Diet: Difficulties Celiac Disease Patients have to Face Daily. Austin J. Nutr. Food Sci..

[14] Araújo H.M.C., Araújo W.M.C. (2011). Coeliac disease. Following the diet and eating habits of participating individuals in the Federal District, Brazil. Appetite.

[15] Silva R.P. (2010). Da Detecção e Quantificação de Glúten em Alimentos Industrializados por Técnica de ELISA.

[16] Laureano Á.M. (2010). Análise da Presença de Glúten em Alimentos Rotulados Como Livres de Glúten Através de Ensaio Imunoenzimático e de Fitas Imunocromatográficas.

[17] Mattioni B., Scheuer P.M., Antunes A.L., Paulino N., De Francisco A. (2016). Compliance with gluten-free labelling regulation in the brazilian food industry. Cereal Chem..

[18] Lee H.J., Anderson Z., Ryu D. (2014). Gluten Contamination in Foods Labeled as “Gluten Free” in the United States. J. Food Prot..

[19] Thompson T., Lee A.R., Grace T. (2010). Gluten Contamination of Grains, Seeds, and Flours in the United States: A Pilot Study. J. Am. Diet. Assoc..

[20] Sharma G.M., Pereira M., Williams K.M. (2015). Gluten detection in foods available in the United States—A market survey. Food Chem..

[21] Oliveira O.M.V., Zandonadi R.P., Gandolfi L., de Almeida R.C., Almeida L.M., Pratesi R. (2014). Evaluation of the Presence of Gluten in Beans Served at Self-Service Restaurants: A Problem for Celiac Disease Carriers. J. Culin. Sci. Technol..

[22] McIntosh J., Flanagan A., Madden N., Mulcahy M., Dargan L., Walker M., Burns D.T. (2011). Awareness of coeliac disease and the gluten status of ‘gluten-free’ food obtained on request in catering outlets in Ireland. Int. J. Food Sci. Technol..

[23] Sainsbury K., Mullan B. (2011). Measuring beliefs about gluten free diet adherence in adult coeliac disease using the theory of planned behaviour. Appetite.

[24] Biagetti C., Naspi G., Catassi C. (2013). Health-related quality of life in children with celiac disease: A study based on the Critical Incident Technique. Nutrients.

[25] Meyer S., Rosenblum S. (2017). Activities, Participation and Quality of Life Concepts in Children and Adolescents with Celiac Disease: A Scoping Review. Nutrients.

[26] BRASIL. Ministério da Saúde. Agência Nacional de Vigilância Sanitária (2004). Resolução RDC n 216, de 15 de Setembro de 2004.

[27] BRASIL. Ministério do Desenvolvimento Social e Combate à Fome (2004). Manual-Programa Restaurante Popular.

[28] Carrijo A., Botelho R., Akutsu R., Zandonadi R. (2018). Is What Low-Income Brazilians Are Eating in Popular Restaurants Contributing to Promote Their Health?. Nutrients.

[29] Instituto Brasileiro de Geografia e Estatística (2011). Pesquisa de Orçamentos Familiares: Análise do Consumo Alimentar Pessoal no Brasil.

[30] Karajeh M.A., Hurlstone D.P., Patel T.M., Sanders D.S. (2005). Chefs’ knowledge of coeliac disease (compared to the public): A questionnaire survey from the United Kingdom. Clin. Nutr..

[31] Cranney A., Zarkadas M., Graham I.D., Butzner J.D., Rashid M., Warren R., Molloy M., Case S., Burrows V., Switzer C. (2007). The Canadian Celiac Health Survey. Dig. Dis. Sci..

[32] Simpson S., Lebwohl B., Lewis S.K., Tennyson C.A., Sanders D.S., Green P.H. (2011). Awareness of gluten-related disorders: A survey of the general public, chefs and patients. e-SPEN Eur e-J. Clin. Nutr. Metab..

[33] Laporte L., Puppin Zandonadi R. (2011). Conhecimento Dos Chefes De Cozinha Acerca Da Doença Celíaca. Alim. Nutr..

[34] Farage P., de Medeiros Nóbrega Y.K., Pratesi R., Gandolfi L., Assunção P., Zandonadi R.P. (2017). Gluten contamination in gluten-free bakery products: A risk for coeliac disease patients. Public Health Nutr..

[35] Simpson S., Thompson T. (2012). Nutrition Assessment in Celiac Disease. Gastrointest. Endosc. Clin. N. Am..

[36] Falcomer A.L., Santos Araújo L., Farage P., Santos Monteiro J., Yoshio Nakano E., Puppin Zandonadi R. (2018). Gluten contamination in food services and industry: A systematic review. Crit. Rev. Food Sci. Nutr..

[37] Bicudo M.O.P. (2010). Avaliação da Presença de Glúten em Produtos Panificados Para Celíacos: Estudo de Caso.

[38] Farage P., Puppin Zandonadi R., Cortez Ginani V., Gandolfi L., Pratesi R., de Medeiros Nóbrega Y.K. (2017). Content Validation and Semantic Evaluation of a Check-List Elaborated for the Prevention of Gluten Cross-Contamination in Food Services. Nutrients.

[39] Petruzzelli A., Foglini M., Paolini F., Framboas M., Serena Altissimi M., Naceur Haouet M., Mangili P., Osimani A., Clementi F., Cenci T. (2014). Evaluation of the quality of foods for special diets produced in a school catering facility within a HACCP-based approach: A case study. Int. J. Environ. Health Res..

[40] Fenacelbra Guia Orientador Para Celíacos. http://www.fenacelbra.com.br/fenacelbra/wp-content/uploads/2013/04/Guia-Orientador-para-Celíacos.pdf.

[41] Zarkadas M., Dubois S., MacIsaac K., Cantin I., Rashid M., Roberts K.C., La Vieille S., Godefroy S., Pulido O.M. (2013). Living with coeliac disease and a gluten-free diet: A Canadian perspective. J. Hum. Nutr. Diet..

[42] Chauhan J.C., Kumar P., Dutta A.K., Basu S., Kumar A. (2010). Assessment of dietary compliance to Gluten Free Diet and psychosocial problems in Indian children with celiac disease. Indian J. Pediatr..

[43] Roma E., Roubani A., Kolia E., Panayiotou J., Zellos A., Syriopoulou V.P. (2010). Dietary compliance and life style of children with coeliac disease. J. Hum. Nutr. Diet..

[44] Whitaker J.K.H., West J., Holmes G.K.T., logan R.F.A. (2009). Patient perceptions of the burden of coeliac disease and its treatment in the UK. Aliment. Pharmacol. Ther..

[45] Rodríguez Almagro J., Hernández Martínez A., Lucendo A.J., Casellas F., Solano Ruiz M.C., Siles González J. (2016). Health-related quality of life and determinant factors in celiac disease. A population-based analysis of adult patients in Spain. Rev. Esp. Enferm. Dig..

[46] Hollon J.R., Cureton P.A., Martin M.L., Puppa E.L.L., Fasano A. (2013). Trace gluten contamination may play a role in mucosal and clinical recovery in a subgroup of diet-adherent non-responsive celiac disease patients. BMC Gastroenterol..

[47] Mena M.C., Lombardía M., Hernando A., Méndez E., Albar J.P. (2012). Comprehensive analysis of gluten in processed foods using a new extraction method and a competitive ELISA based on the R5 antibody. Talanta.

